# Complex tools and motor-to-mechanical transformations

**DOI:** 10.1038/s41598-022-12142-3

**Published:** 2022-05-16

**Authors:** M. Ras, M. Wyrwa, J. Stachowiak, M. Buchwald, A. M. Nowik, G. Kroliczak

**Affiliations:** 1 Action and Cognition Laboratory, Faculty of Psychology and Cognitive Science, Adam Mickiewicz University, ul. Szamarzewskiego 89, 60-568 Poznan, Poland; 2 Faculty of Psychology and Cognitive Science, Adam Mickiewicz University, Poznan, Poland

**Keywords:** Motor control, Sensorimotor processing

## Abstract

The ability to use complex tools is thought to depend on multifaceted motor-to-mechanical transformations within the left inferior parietal lobule (IPL), linked to cognitive control over compound actions. Here we show using neuroimaging that demanding transformations of finger movements into proper mechanical movements of functional parts of complex tools invoke significantly the right rather than left rostral IPL, and bilateral posterior-to-mid and left anterior intraparietal sulci. These findings emerged during the functional grasp and tool-use programming phase. The expected engagement of left IPL was partly revealed by traditional region-of-interest analyses, and further modeling/estimations at the hand-independent level. Thus, our results point to a special role of right IPL in supporting sensory-motor spatial mechanisms which enable an effective control of fingers in skillful handling of complex tools. The resulting motor-to-mechanical transformations involve dynamic hand-centered to target-centered reference frame conversions indispensable for efficient interactions with the environment.

## Introduction

While many species in the animal kingdom possess neural and cognitive mechanisms enabling tool use^1^, the abilities of humans are exceptional. Monkeys, for example, train for many months to engage their sensorimotor intelligence in efficient handling of complex tools, where the hand holding a tool performs movements different from the ones by the functioning tool parts^2^. Perhaps such compound *motor-to-mechanical* transformations^3^ are too intricate to be easily executed by the monkey brain. Consistent with this notion, only in humans does the rostral part of the inferior parietal lobule (IPL) in the left hemisphere respond to observation of tool use actions^4^, even when the tools just extend or amplify the acting hand. Whether or not left IPL, one of the most expanded and lateralized association regions in the human brain^5,6^, is the seat of the ability for skilled use of complex tools is still an open question.

The control of actions through the postulated transformations from motor to mechanical codes^7^ is unlikely to rely just on left IPL. In fact, there is some evidence that it is orchestrated within the left-lateralized temporo-parieto-frontal *praxis representation network* (PRN)^3,8^, with IPL as a critical node. Yet, prior investigations on the neural control of tool use exploited actions involving mainly simple tools. For such tools, movements of the hand or its fingers and movements of tool parts are equivalent^9,10^, and the neural underpinnings involved in their control cannot be distinguished. Meanwhile, the only two studies which utilized actions with a complex tool (i.e., a gripper) supporting the grasping hand^11,12^, compared the neural control of those actions to grasping with bare hand. Therefore, the differences between IPL contributions to the control of *proximal* motor outputs—i.e., hand centered guidance^13^ of the grasped tool, and the control of *distal* mechanical outputs—i.e., target centered guidance of functional tool parts^2^ still remain unknown. Interestingly, for disparate classes of complex tools, the required motor-to-mechanical transformations are not straightforward or limited to grasping actions (e.g., riding a reverse steering bicycle is impossible without training). Yet, the human brain is capable of implementing them efficiently and faster than the monkey brain. Given the differences between species, a natural question is where and when such implementations take place, and what cognitive mechanisms are invoked.

Here, we used functional magnetic resonance imaging (fMRI) to measure neural activity associated with preparation and execution of complex tool use actions, involving multifaceted motor-to-mechanical transformations evaluated in a multi-step paradigm. It consisted of three phases: (1) the planning of functional grasps, including prospective kinematics for grasping relevant tool parts, e.g., handles; (2) performance of functional grasps and immediate programming of pertinent hand/finger movements and their timing (achieving proximal goals) for later usage of tools, and (3) the physical performance of actions involving such tools while acting on intended targets (achieving distal goals). Because the last phase would involve primarily monitoring of the visuo-motor feedback on the success of the ongoing task, the most critical computations should take place either during the grasp planning phase^12,14^ or the grasping performance phase^15^, comprising time intervals when the tool is held in hand, and the expected functional movement kinematics are programmed. We used real complex and simple tools, and target objects (*recipients*) to be acted on or interacted with, because motor-to-mechanical transformations should not be just simulated, as shown by actual and imagined riding the reverse steering bicycle. Indeed, such actions require physical objects, providing sensory feedback to be fully implemented. We focused on the engagement of left IPL and IPS, regarded as key for tool use actions, especially in humans^4,16^.

**Figure 1:**
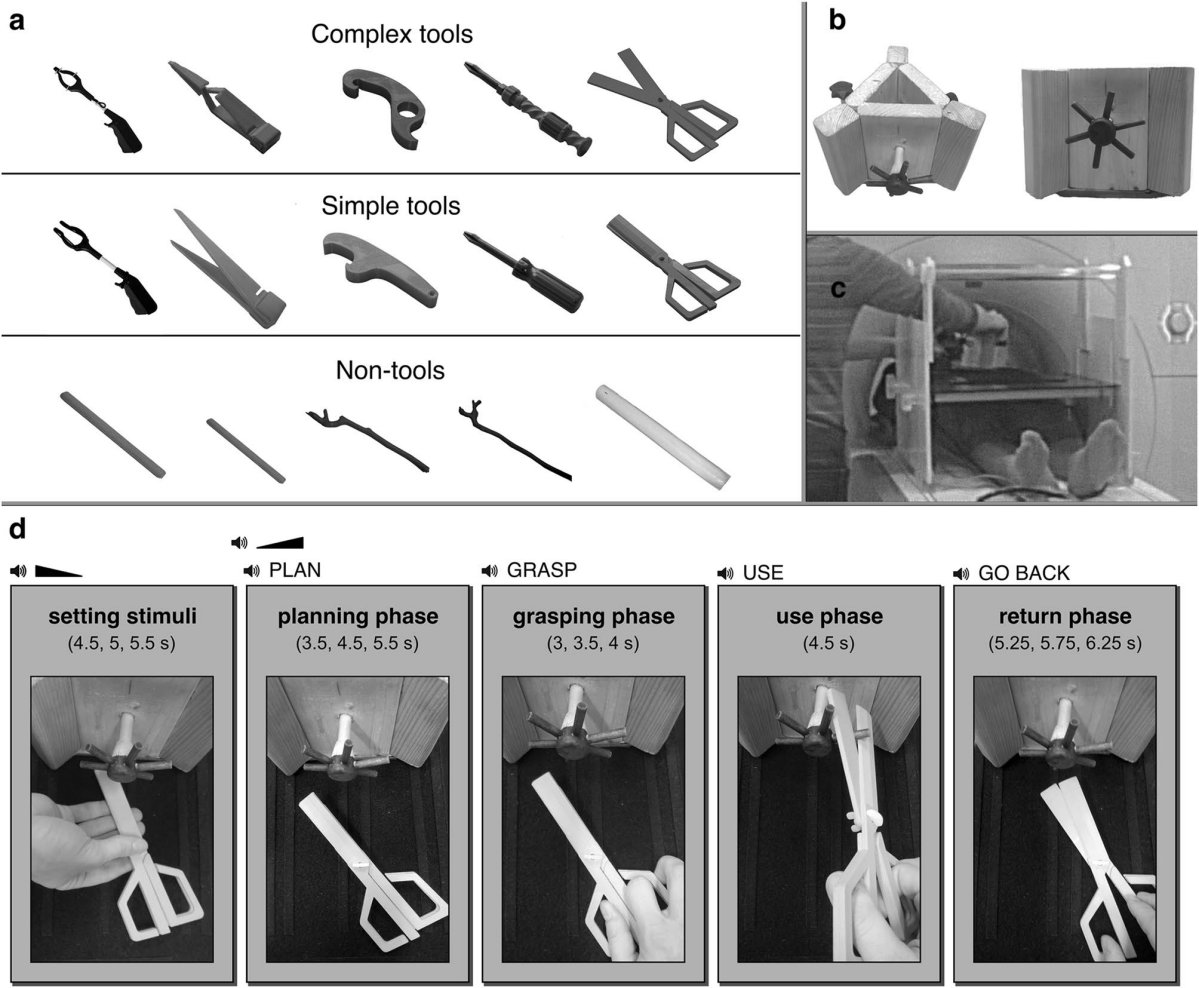
Stimuli, apparatus and study design. (**a**) Examples of stimuli. Two sets of complex, and simple tools in two different sizes, as well as one set of control objects was utilized. (**b**) Action recipients. We devised three recipient objects to be acted on by all stimulus kinds. (**c**) Stimuli and action recipients were positioned on the apparatus table by an experimenter before trial onset. (**d**) Trial structure and timing of tasks in main experiments. We used an event-related design in which different events had variable durations, as depicted within each frame at the bottom of this figure. Participants could view the workspace only in time intervals indicated by the auditory cue. Eyes were always closed during stimulus set up intervals.

To maximize a chance of invoking critical parietal mechanisms characterizing human sensorimotor intelligence but spontaneously inaccessible in monkeys, we created several, fMRI *magnet friendly*, complex tools that allowed dissociation of lower-level kinematics of the hand and fingers handling such implements, and the workings of their functional parts prior to and when in contact with intended targets. Figure 1a shows one set of such tools, i.e., a gripper, tweezer, opener, rotational screwdriver, and flipped scissors, whose manipulations—during grasping or holding, opening, driving, or cutting—require proximal finger/hand movements opposite to or different from distal movements of functional tool parts, an imperative for the aim of this study. After all, we wanted to disclose the neural mechanisms involved in higher-order control of complex tools, rather than in the control of finger muscles^2^. Our complex tools are shown together with their common counterparts which are simple tools that just extend and amplify the hand and fingers during their usage. Each set of tools consisted of two versions of different sizes so that participants could not easily get used to manipulating them. Figure 1a also shows our control objects, e.g., sticks and twigs with no clear functions.

During action performance, the tools were directed at real targets (shown in Fig. 1b), adjusted by the experimenter (Fig. 1c) in accordance with a function tested on a given trial. The control objects were used only for reaching out and repeatedly touching their targets. Figure 1d also illustrates five events making an individual trial including those critical for our three-stage paradigm. The variable durations of each trial with a tool in distinct phases, i.e., the planning of functional grasp, grasp execution with tool-use action programing, and performance of target directed tool use, are shown together with an example tool, and relevant responses. Analogical phases with variable durations were also introduced for trials with control objects. Our approach is consistent with the premise that complex actions are naturally parsed into temporo-spatial chunks controlled by disparate neural and cognitive mechanisms^17^.

To identify brain areas modulated by motor-to-mechanical transformations inherent to tool-related actions, we used whole-brain equally weighted contrasts of tasks involving both tool categories with non-tool control objects. We carried out these background comparisons separately for all three stages of task performance, that is for planning, grasping, and using, to shed light on the outcomes of the main tests—direct and critical contrasts of complex and simple tools in these same performance stages. The background outcomes would let us know whether or not the areas/neural mechanisms modulated by the implementation of complex motor-to-mechanical transformations belong to the praxis network or are outside of it, especially in IPL. We were particularly interested in identifying phase-related contingencies of direct comparisons between tasks involving complex and simple tools, i.e., when such transformations take place. Finally, to be consistent with neuropsychological traditions, wherein in patients with left-hemisphere damages the non-hemiparetic (or non-paralyzed) left hands are typically examined to reveal higher-order, hand-independent representations of motor skills^18,19^, we tested both the dominant right, and non-dominant left hand. By using hand as a factor in analyses, we not only increased the power of our tests but also maximized our chances of finding the hand-independent substrates of human sensorimotor intelligence^1^.

## Results

### Right IPL is involved in motor-to-mechanical transformations during the transition from grasping to using of complex tools

**Figure 2:**
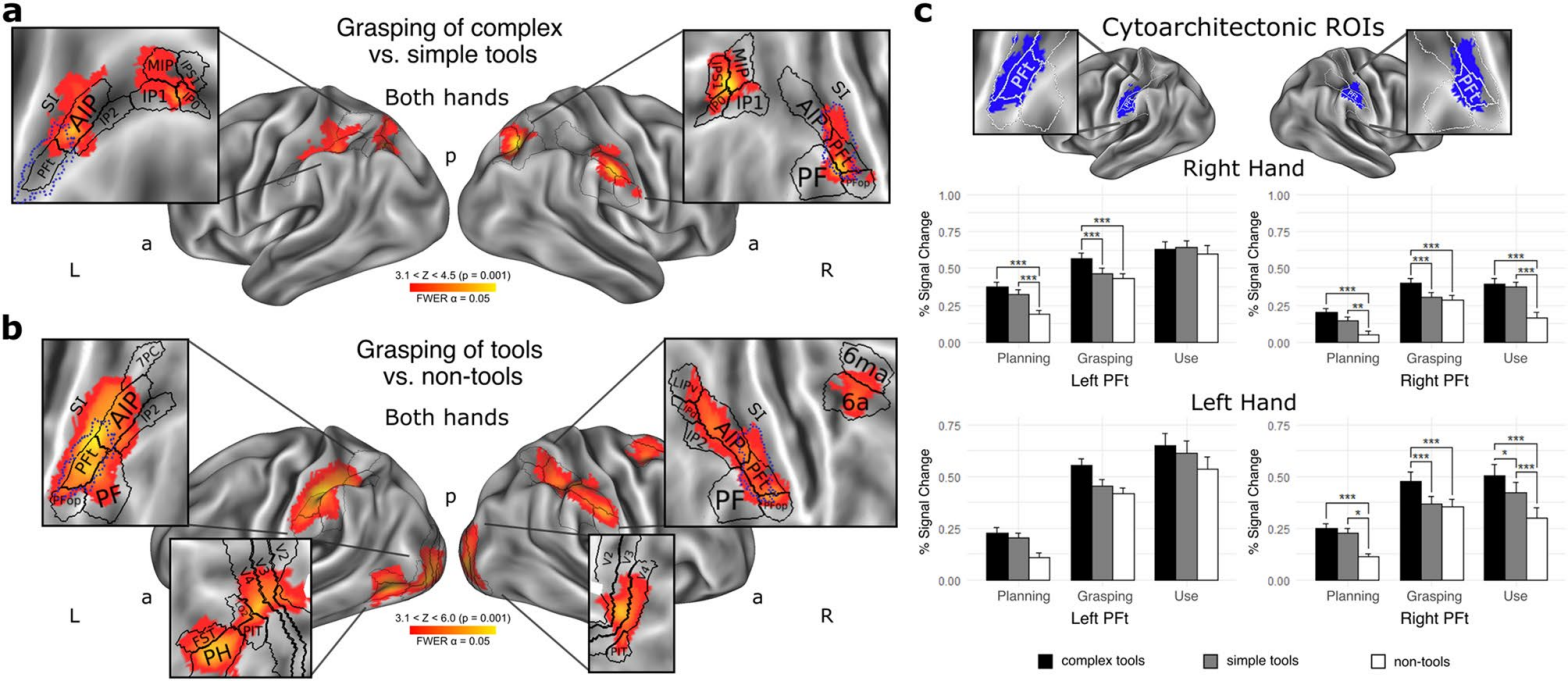
Neural correlates of motor-to-mechanical transformations. (**a, b**) Task-related contrasts revealing significantly different clusters of voxels, with each voxel thresholded at Z > 3.1, and a (corrected) cluster significance threshold of p = 0.05 (controlling for family-wise error rate, FWER), during the transition from grasping to using of (**a**) complex tools as compared to simple tools, and (**b**) both simple and complex tools, as compared to control objects, i.e., sticks and twigs, regardless of the hand. Insets with flattened brain surfaces depict significantly active areas in more detail (by the use of parcels from an atlas^23^). Blue dots in insets indicate smoothed borders of the Region of Interest (ROI) shown in (**c**). (**c**) ROI analyses in the independently defined *tenuicortical* supramarginal area (PFt) of the inferior parietal lobule (based on Juelich probabilistic cytoarchitectonic maps, thresholded at 50th% of their maximum probability^21^) and visualized in top row on the right. Middle row: graphical depiction of the results of a significant interaction of phase (Planning, Grasping, Using) and object category (Complex tools, Simple tools, Non-tools) in the 3 × 3 repeated-measures analysis of variance (rmANOVA) for the left PFt (F_2.447, 46.496_ = 5.999, P = 0.0028, ηp^2^ = 0.240, 1-β = 0.906) and right PFt (F_4, **7**6_ = 6.695, P = 0.0001, ηp^2^ = 0.261, 1-β = 0.990) conducted on neural signals associated with tasks performed with the dominant right hand. Bottom row: this same interaction observed in the left (F_4, **7**6_ = 1.645, P = 0.172) and right PFt (F_4, **7**6_ = 4.310, P = 0.0034, ηp^2^ = 0.185, 1-β = 0.915) for tasks performed with the non-dominant left hand. Asterisks indicate significant differences with p-values of ≤ 0.05 (*), 0.01 (**), or 0.001 (***) in post hoc Bonferroni corrected t-tests.

Out of the three task phases tested here, only in the grasping phase followed immediately by preparation for tool-use actions—i.e., the time interval for the programming of finger and hand movements, we found significantly greater activity for complex tools contrasted with simple tools. This effect was revealed by a 2 (hand: right, left) × 2 (tool category: complex, simple) repeated-measures analysis of variance (rmANOVA), utilizing rest intervals as baseline; Z > 3.1, P = 0.001, FWER α = 0.05^20^). Specifically, as depicted in Fig. 2a, we observed significantly greater engagement of the right rostral inferior parietal lobule (rIPL), with peak activity in the *tenuicortical*, that is rostral subdivision of the anterior supramarginal gyrus (aSMG), namely its PFt parcel, and the cluster extended antero-ventrally to areas PF and PFop, postero-dorsally to AIP, and anteriorly to the primary somatosensory cortex (SI, specifically area 2), as corroborated by neuroanatomical, cytoarchitectonic, and multimodal parcellation atlases^21–24^. Counter to our expectations, there was no comparable activity in left rIPL. Instead, this contrast revealed significantly greater engagement of the left anterior intraparietal sulcus (aIPS), with peak activity in the multimodal AIP parcel, and the cluster extended weakly to aSMG (mainly PFt) and lateral IP2, as well as dorsally to area 7PC, and again even to SI (area 2). Finally, our main contrast of complex vs. simple tools, in the grasping/tool-use programming phase, disclosed bilateral posterior-to-mid IPS contributions, including such subdivisions as medial intraparietal (MIP), IP1, and IP0 parcels, as well as IPS1 exclusively on the right. Similarly to right rIPL, the right-hemisphere IPS areas were also more sensitive to the difference between complex and simple tools.

A background contrast of tools vs. non-tools performed in the same grasping/tool-use programming phase revealed significant tool-specific parietal neural activity limited only to aSMG and aIPS. As Fig. 2b shows, the contribution of the left hemisphere was greater but more focal, and limited mainly to subdivisions PFt and AIP (with an activity peak located at their borders), with the cluster extending to areas IP2, PF, and PFop (and starting from here, also along the sulcal borders of area 2 to area 7PC). Conversely, there was little SI contribution in the right hemisphere, and it was limited only to the intersection of area 2 with AIP and PFt. Yet, the cluster extended more posteriorly to the lateral intraparietal (LIP) subdivisions, both dorsal and ventral (LIPd and LIPv, respectively). We also found a significantly greater, tool-specific activity in the lateral occipitotemporal cortex (LOTC; involving areas PH, FST, PIT, LO2) on the left, as well as bilateral contributions from lower-level visual areas. Interestingly, this same contrast (tools vs. non-tools) showed some unexpected contributions from the right dorsal premotor and supplementary motor area (6a, 6ma).

Only the region of interest (ROI) analysis^25^ partly revealed an effect consistent with our hypothesis and indicated that left rIPL, i.e., an independent, cytoarchitectonically defined PFt subdivision, showed greater engagement for complex tools, as directly compared to simple tools (Fig. 2c). Similarly to the whole brain analysis (Fig. 2a), we found this effect in the grasping/tool-use programming phase. Critically, for the dominant right hand, the pattern of neural activity changes in left PFt ROI was the same as in the right PFt ROI. Interestingly, there was no difference between simple tools and non-tools in the two ROIs in the grasping phase. Furthermore, and also consistent with the whole brain analyses depicted in Fig. 2a, there was no difference between complex and simple tools observed in the initial planning nor later using phase (and therefore not shown in Fig. 2, as the whole-brain contrasts for these two phases were empty). Yet, significant differences between the two categories of tools and control non-tool objects were observed in both left and right PFt during grasp planning, and only in right PFt for tool use execution (Fig. 2c, middle two panels on the right).

For the non-dominant left hand (Fig. 2c, bottom two panels on the right), in the grasping phase we found no difference between complex and simple tools in left PFt (consistent with the whole-brain results shown in Fig. 2a). Yet, similarly to the dominant right hand (Fig. 2c, middle panel on the right), there were significant differences in right PFt. Notably, the difference between complex and simple tools now also extended to the tool use phase. In the right PFt, there was also a familiar difference between tools and control objects in the planning phase, a familiar lack of significant difference between simple tools and non-tools in the grasping phase, but in the use phase we found significant differences between all three categories of objects, with the greatest activity observed for complex tools.

The absence of the expected greater contribution of left rIPL to the control of complex tools in the whole brain analyses (as indicated by empty contrasts for the planning, and using phase, and little effect observed in left PFt in the grasping phase, see Fig. 2a), and its appearance in the ROI analysis for the right hand prompted us to further investigate this issue. Additional higher-level mixed-effects variance estimations based on Metropolis–Hastings Markov Chain Monte Carlo sampling (revealing near-threshold voxels as potentially significant, were our sample size substantially larger) were used to further approximate parameters for higher-level contrasts^26^ at the hand independent level. They revealed some engagement of the left rIPL, too. Yet, the additional significantly active voxels were located mainly in subdivisions PF and PFop, rather than in PFt, as shown in Supplemental Fig. 1a. This same figure also illustrates that virtually identical results were obtained in the right hemisphere. Interestingly, the same type of analysis performed separately for the dominant right (Supplemental Fig. 1b) and non-dominant left hand (Supplemental Fig. 1c) indicated that additional rIPL and further processing elsewhere in the brain may play a greater role in the control of the non-dominant hand. Furthermore, as Supplementary Fig. 1d shows, the main effect of tool category—i.e., complex vs. simple—that we obtained regardless of the analysis type for the grasping/tool-use programming phase, was largely hand independent. Specifically, the network of areas revealed by contrasting complex and simple tools in the grasping phase using additional Bayesian modeling and estimation does not overlap with the activity shown by a main effect of hand. Therefore, these outcomes emphasize the hand-independence of main results of this study. In other words, the identified significant clusters were located mainly outside of the hand-dependent neural activity (see Supplemental Fig. 1d for details).

**Figure 3:**
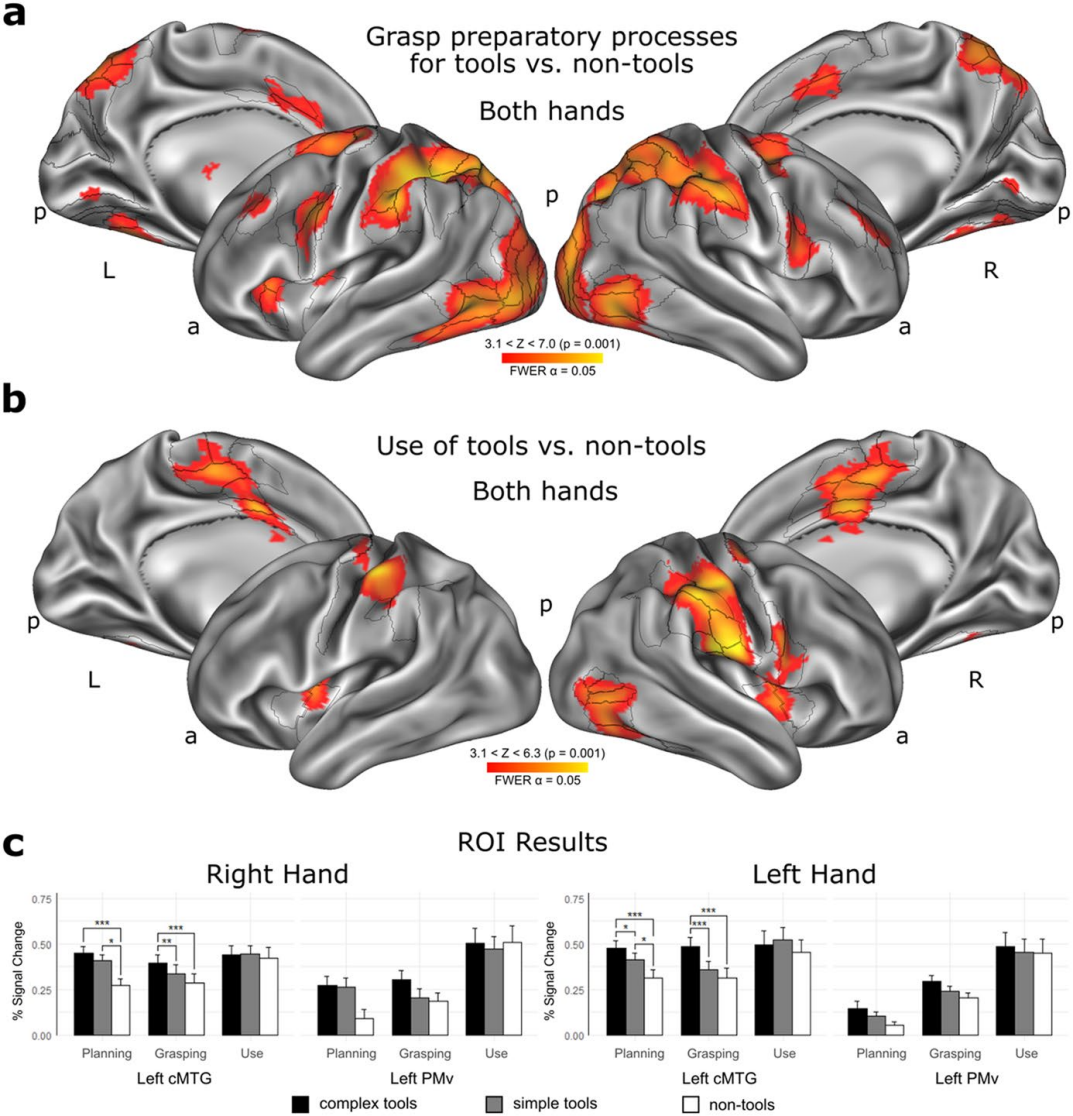
Brain areas involved in planning, grasping and using of complex and simple tools. (**a**, **b**) Task-related contrasts revealing significantly different clusters of voxels, thresholded at least at Z > 3.1, and a cluster-corrected (family-wise error rate, FWER controlled) significance threshold of p ≤ 0.05, for (**a**) planning functional grasps of tools (grasp preparatory processes) contrasted with non-tools, and (**b**) tool use contrasted with pointing movements with non-tools, regardless of the used hand. (**c**) ROI (region of interest) analyses in the left caudal middle temporal gyrus (cMTG) and ventral premotor cortex (PMv), with percent signal change (%SC) extracted from 5-mm dimeter spheres centered at coordinates of local signal peaks obtained from an independent tool use localizer (see Methods). A 3 × 3 repeated-measures analysis of variance (rmANOVA) in left cMTG revealed a significant phase (Planning, Grasping, Using) by object category (Complex tools, Simple tools, Non-tools) interaction for both tasks performed with the dominant right hand (F_4,76_ = 3.491, P = 0.0113, ηp^2^ = 0.155, 1-β = 0.841) and the non-dominant left hand (F_2.757,52.375_ = 4.412, P = 0.0093, ηp^2^ = 0.188, 1-β = 0.827). This same analyses conducted for left PMv revealed neither significant interaction for tasks performed with the right hand (F_2.054,39.023_ = 2.877, P = 0.0670) nor the left hand (F_4,76_ = 0.608, P = 0.658), although a similar trend was observed only for the dominant hand. Asterisks indicate significant differences with p-values of ≤ 0.05 (*), 0.01 (**), or 0.001 (***) in post hoc Bonferroni corrected t-tests.

For completeness, it should be also stated that all clusters showing significantly greater activity for complex tools in the grasping/tool-use programming phase (Fig. 2a) were typically found within the confines of the praxis network revealed by contrasting tools, regardless of whether complex or simple, and control sticks or twigs used for pointing or touching the target objects. This network shown in Fig. 3a was rather symmetrical in its extent but nevertheless engaged more in the left hemisphere (see also Fig. 2c). Yet, the most reliable difference observed for grasping complex vs. simple tools was in the right rIPL, not the left one. Consistent with its special, though so far unappreciated, role in controlling actions involving tools is the effect shown in Fig. 3b for the tool-use execution phase, wherein it was right rIPL in collaboration with aIPS and SI that contributed most to effective guidance of tools towards their recipient targets, and actions on them. These converging outcomes—pointing to right SMG, and its PFt subdivision in particular—indicate that this parietal area plays a key function in programming and coordinating hand/digit movements for compound manual actions involving tools. Table 1 shows coordinates of peak activity, and their values in regions with significant involvement revealed by the two major fMRI contrasts from the main experiment.

**Table 1:** Peak Montreal Neurological Institute (MNI) coordinates and peak values of the regions exhibiting significant activity in two major contrasts from the main experiment. For each area/cluster identified with a given contrast, MNI coordinates of local peaks and maximal Z values are reported. These clusters were obtained using the modeling of random-effects components of mixed-effects variance and thresholded at Z > 3.1, p = 0.001, with family-wise error rate (FWER) corrected at α ≤ 0.05.

	MNI Coordinates			Peak value z-max
	x	y	z	
**(A) Grasping of complex versus simple tools**				
Left Intraparietal Sulcus, anterior Intraparietal Area, AIP	− 42	− 38	42	4.33
Left Anterior Parietal Lobe, Superior Parietal Lobule, 2/7PC	− 38	− 42	54	3.51
Left Intraparietal Sulcus, medial Intraparietal Area, MIP	− 22	− 60	48	4.39
Left Intraparietal Sulcus, posterior Intraparietal parcels, IP1/IP0	− 30	− 72	36	3.96
Right Inferior Parietal Lobule, anterior Supramarginal Gyrus, PFt	62	− 20	40	4.3
Right Intraparietal Sulcus, anterior Intraparietal Area, AIP/2	42	− 30	40	3.71
Right Intraparietal Sulcus, mid-to-posterior Intraparietal parcels, MIP/IPS1/IP0	28	− 64	36	4.58
**(B) Grasping of tools versus non-tools**				
Left Intraparietal Sulcus, anterior Intraparietal Area, AIP	− 40	− 32	36	6.2
Left Inferior Parietal Lobule, anterior Supramarginal Gyrus, PFt	− 54	− 28	38	5.32
Left anterior Parietal Cortex, Postcentral Gyrus, 2/PFop	− 62	− 20	38	5.13
Left Occipital Cortex, Occipital Pole, V4v	− 36	− 92	− 6	6.07
Left Occipital Cortex, Lateral Occipital Cortex, PH	− 46	− 66	− 6	5.55
Right Inferior Parietal Lobule, anterior Supramarginal Gyrus, PFt	62	− 18	32	4.67
Right Intraparietal Sulcus, anterior Intraparietal Area, AIP/PFt	50	− 30	44	4.35
Right Frontal Cortex, Superior Frontal Sulcus, 6a	26	0	56	4.34
Right Frontal Cortex, Superior Frontal Gyrus, 6ma	20	4	62	3.74
Right Occipital Cortex, Occipital Pole, V3v	34	− 92	2	5.47
Right Occipital Cortex, ventro-lateral Occipital Cortex, PIT	40	− 84	− 10	4.06

#### ROI analyses performed outside of the parietal lobe

These analyses corroborated that the left caudal middle temporal gyrus (cMTG, including subdivisions PH, FST, and PHT, regardless of the hand), an area also belonging to PRN (the praxis representation network), revealed a familiar simple main effect of tool category during the grasping phase, too, as corroborated by post hoc testing for the significant phase by object type interaction. Figure 3c demonstrates that the effect was present for both hands and, somewhat surprisingly, in the planning phase for the left hand. Nevertheless, consistent with the prior results, during the planning phase both tool categories engaged cMTG more than the control objects. Finally, counter to all previously reported results, the left ventral premotor cortex (PMv, including area 6r and 6v) showed no familiar interaction between study phase and object type category, despite some trends towards significance for the right hand.

#### Motor-to-mechanical transformations in response time patterns

*In scanner testing*. The execution of grasping actions performed towards target objects during fMRI experiments was, unexpectedly, associated with movement onset times for tools, regardless of whether complex or simple, that were faster than for non-tool objects. Specifically, as revealed by a 2 × 3 rmANOVA, with hand (right, left) and object type (simple tools, complex tools, non-tools) as within-subjects factors, movement onsets for grasping complex tools were 23 ms faster (Bonferroni corrected P = 0.01) and for simple tools were 16 ms faster (P = 0.029) than for non-tools, regardless of the used hand. (Only a main effect of object type was significant, F_1.547,29.402_ = 8.82, P = 0.002, ηp^2^ = 0.317, 1-β = 0.916.) Because these outcomes suggested that there are potential differences in movement onsets for grasping complex and simple tools, too, we conducted an additional behavioral experiment outside of the scanner using a different sample of participants. We measured both response times (movement onsets, as in the main experiments) and grasp kinematics. Because there was no hand effect observed in the outcomes from our fMRI study, we focused only on testing the dominant hand.

##### Outside of the scanner testing

**Figure 4:**
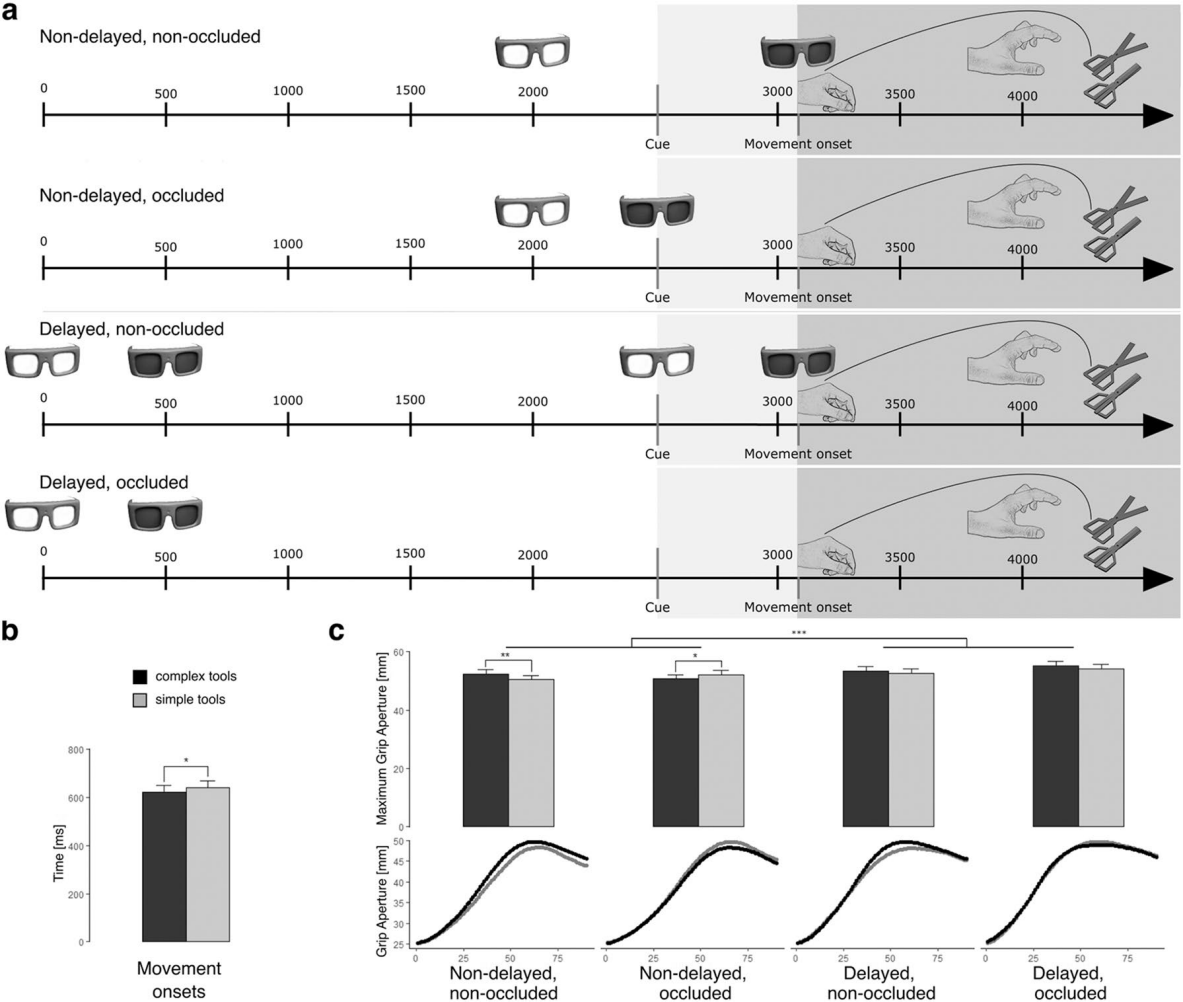
Trial structure, timing, and results from the experiment on grasping complex and simple tools outside of the neuroimaging scanner. (**a**) Four kinds of trial types based on the availability of vision (preview: occluded, non-occluded) and time to the start cue (trial type: delayed, non-delayed) were introduced. For simplicity, they are aligned with the start cue. Tools, either complex or simple, were always presented for at least 0.5 s. Grasping was executed either with no delay (top two rows) or after a 2-s delay (bottom two rows). In trials with non-occluded vision, programming of grasp kinematics, following the start cue, continued with vision available, and was blocked simultaneously with movement onset. Hence, grasping was always performed without visual feedback. In trials with no delay but occluded preview, vision was blocked with the start cue. In delayed and non-occluded trials, following a 2-s interval with no vision available after initial preview, vision was restored with the start cue, and again blocked with movement onset. In delayed and occluded trials, vision was blocked after 0.5-s preview and never restored. Then, grasping triggered by the start cue was performed exclusively based on remembered tool image. We measured movement onsets following to the start cue, and maximum grip aperture (MGA) before target tools were grasped. (**b**) Results for movement onsets for grasping complex and simple tools. Participants initiated grasping movements significantly faster for complex tools. (**c**) Grasp kinematics. Top row: MGAs for complex and simple tools contingent on trial type (non-delayed, delayed) and preview (non-occluded, occluded). We found a significant trial type × preview × tool category interaction (see main text for details) wherein MGAs in trials with non-occluded preview and executed with no delay were significantly larger for complex tools, whereas in trials executed following a delay MGAs were significantly larger for simple tools.

Figure 4a shows the paradigm that we used for testing grasp movement onsets and grip apertures before contact with complex and simple tools, and Fig. 4b and Fig. 4c depict the main results. We found that a 2 (tool category: complex, simple) × 2 (trial type: no-delay, delay) × 2 (preview: non-occluded, occluded) rmANOVA revealed a significant main effect of tool category (F_1,15_ = 7.420, P = 0.0155, ηp^2^ = 0.331, 1-β = 0.723), and it was such that movement onsets towards complex tools were reliably faster than the ones towards simple tools (a mean difference of 19 ms, shown in Fig. 4b). None of the remaining factors interacted with tool complexity and, therefore, a significant main effect of trial type, and preview, as well as a significant trial type × preview interaction is in the Supplementary materials.

### Motor-to-mechanical transformations in movement kinematics

We found that a 2 × 2 × 2 rmANOVA with the same between-objects and within-subjects factors revealed only a significant main effect of preview (non-occluded, occluded) before movement onsets (F_1,15_ = 27.942, P = 0.000091, ηp^2^ = 0.651, 1-β = 0.998), such that participants’ maximal grip apertures (MGAs) were substantially larger in trials with vision occluded prior to grasp execution (MD = 2.45 mm). Counter to response time data, the expected main effect of tool category (complex, simple) was not significant (F_1,15_ = 1.715, P = 0.210). There was, however, a significant interaction of tool category with trial type (no-delay, delay), F_1,15_ = 6.70 (P = 0.021, ηp^2^ = 0.309, 1-β = 0.677), wherein participants’ MGAs for complex tools were significantly larger only in no-delay trials (MD = 1.23 mm, P = 0.0166). Finally, we also found a significant trial type × preview × tool category interaction (F_1,15_ = 7.32, P = 0.0163, ηp^2^ = 0.328, 1-β = 0.715) wherein MGAs in trials with non-occluded preview and executed with no delay were larger for complex tools (MD = 1.78 mm, P = 0.0028), whereas in trials executed following a delay MGAs were larger for simple tools (MD = 1.38 mm, P = 0.0453). These effects are illustrated in Fig. 4c. There were no further significant effects, although there were trends towards significance for factors unrelated to tool use itself: a trial type (F_1,15_ = 4.172, P = 0.059), and an interaction of trial type and preview (F_1,15_ = 3.482, P = 0.0817). Finally, a two-way interaction of tool category and preview was not significant, either (F < 1.5).

## Discussion

A major scientific challenge in research on tool use abilities has been to show the brain mechanisms involved in skilled control of complex tools^2^. We found that in the human brain the use of such tools, which requires compound transformations of finger movements into disparate mechanical movements of their functional parts, is associated with greater engagement of the right rostral inferior parietal cortex. The contribution of this structure, supporting a computational process also referred to as *distalization* of end effectors^7^, was revealed during grasping of tools and the immediately following tool-use programming phase. These unique results add to an overwhelming body of evidence that it is the left inferior parietal lobule by which the human brain controls skilled use of tools (*praxis*) in a typically organized brain, regardless of the hand, and handedness^4,27^. Critically, the revealed right-hemisphere neural substrates underlying transformations from motor to mechanical codes^7^ can be linked to prospective coordination of fingers movements, based on visual feedback from functional tool parts. (The latter are positioned disparately than the fingers and are expected to act in the opposite or different manner.) These findings are inconsistent with earlier models based on monkey neurophysiology^7^, but are not incohesive with human-based models of functional lateralization in the brain^28,29^. Our study even points to one specific anterior subdivision of the supramarginal gyrus, namely area PFt, missing in earlier monkey neurophysiology models^7,30^. This typically human area^16^ emerged via expansion of the left hemisphere partly due to pressure on development of tool production and tool use skills^6,31,32^. Yet, here we have identified and emphasize a new role of the right PFt in controlling/using complex tools in humans.

### Tool use and the left hemisphere

The left cerebral cortex, and rostral subdivisions of the posterior parietal cortex (PPC) in particular, are key for tool use skills^18,33,34^. Importantly, the emphasis so far has been on the whole supramarginal gyrus^8,35^, or the contribution of its subdivisions (i.e., PF, and PFm) either to manipulation knowledge^36,37^ or technical reasoning, and mechanical problem solving^38,39^. Of course, to fulfill its critical functions, the left supramarginal gyrus must interact with other areas performing diverse neural computations^40,41^, be it perception or action related^3,27,42–45^. Earlier research has demonstrated that areas along the dorso-medial parietal cortex (e.g., subdivisions V6Av, V6Ad, as well as the more anterior PEc or its human equivalent, hPEc) are engaged in planning grasping actions both in the macaque^46^ and human brain^47,48^. Indeed, this cortical vicinity supports numerous complex visuomotor transformations for object directed manual responses, including sensorimotor transformations for performed (not only planned or imagined) actions^49^. Consistent with this notion, the outcomes of our ROI analyses corroborate that the nearby left parietal nodes (e.g., IPS1, IPS3) are engaged more for complex tools in the planning of functional grasp and grasping phase itself, especially for the left hand, putatively requiring more deliberate responses. Yet, because humans, as compared to other species, excel in using disparate varieties of tools, they must have possessed a specialized brain region that allows them to control critical aspects of actions involving complex tools in a more automatic manner. The left rostral IPL seemed a likely candidate because, only in humans, it responds to activities with simple tools which extend and/or amplify the acting hand^4,16^.

For this study, inspired by an earlier project in monkeys trained to use complex gripers^2^, we designed a larger set of complex tools for a greater range of actions, not only limited to grasping food or food-like items^12,50^. We show that the usage of such tools requires predictive coding^51^ of motor-to-mechanical transformations for actions such as grasping, opening, driving, or cutting. These tasks require an orchestration of finger movements of the acting hand with actions exerted by functional tool parts^7^ as there is no direct relation between the two. To reveal their neural underpinnings, we utilized a multi-step paradigm and demonstrated that the greater IPL contribution emerged consistently in the grasping/tool-use programming phase, rather than during grasp planning or tool use phase. Unexpectedly, it was the right PFt that was invariably more invoked for tasks involving complex tools, regardless of the used hand, and analysis type.

### Motor-to-mechanical transformations and the posterior parietal cortex

In the grasping/tool-use programming phase for complex tools, consistent with earlier reports that PFt interacts with other PPC areas^16,49^, we observed greater neural activity in several subdivisions of both the right and left PPC. Thus, in addition to right PFt, our study revealed a right-hemisphere cluster belonging mainly to IPS1 and MIP, and its left-hemisphere counterpart, also involving MIP, but extending to IP1 and IP0, instead. Both in the monkey and human brain, these vicinities are linked to the control of direction of planned movements, and encoding action parameters for the hand, even if movements are self-generated^52,53^. Because graspable parts of complex and simple tools were either identical or well matched, the critical difference here would be an effective, hand-centered or egocentric guidance of the grasping fingers, given the expected mismatch that must be taken into account while programming their movements for the use of complex tools. Consistent with this view are our two further observations. In the grasping experiment performed outside of the fMRI scanner, participants initiated their movements faster, and opened their grips wider for complex tools when vision was available during movement programming. Moreover, when neural activity associated with grasping both categories of tools was contrasted with control objects, no comparable differences in brain functioning were observed in MIP or its vicinity. Apparently, the processing of large dissimilarities in external targets for grasping movements and forthcoming usage is more critical than processing subtle differences in self-generated or egocentric encoding of proximal motor responses for matched tools.

The emphasis on target-centered or the so-called *allocentric* grasp/prospective tool-use encoding in a contrast of tools with non-tools revealed, however, greater bilateral engagement of area PFt. Notably, its activity extended to all abutting neighbors (IP2, AIP, PF, PFop, area 2), also showing the expected advantage of the left-hemisphere processing for tools. This processing was accompanied by neural activity in the latero-ventral perceptual stream starting from V1-V4, via LO2/PIT, through PH/FST^3,14,54^, and partially extending to MST^55^. Areas in this vicinity are modulated by extra-retinal signals in both monkey^55^ and human^43^ studies, and are critical for the storage and retrieval of action concepts^56,57^ and visual processing of tool shapes^14,58^. A greater engagement of the left cMTG ROI for complex tools (regardless of the hand in the grasping phase) can, therefore, be linked to a higher activation of prior knowledge on tools and their usage in order to invoke new action concepts and tool related skills. While all the effects discussed so far emerged within the confines of the praxis network disclosed during the earliest, planning phase for interactions with tools, the actual use of tools revealed greater right-hemisphere engagement of area PFt, and the neighboring IPL, IPS, and SI areas.

Taken together, these pieces of evidence indicate that motor-to-mechanical transformations for proper usage of complex tools are carried out primarily within the right PPC. Their two critical hubs are area MIP and PFt. While right MIP elaborates mainly self-generated or egocentric encoding of proximal hand movements, right PFt is invoked primarily for target-centered or allocentric grasp encoding, and predictive coding of finger movements for their conversion into proper functioning of distal tool parts. MIP and PFt on the right are then critical for effective realization of the intended actions on target objects with the use of complex tools. Yet, because more general tool use skills, and action concepts are stored in the left hemisphere^18,27,59^, this right-sided circuit is expected to closely collaborate with its left-hemisphere counterparts, and other critical areas involved in representing praxis skills^28^.

### The right PFt, and monkey inabilities to spontaneously use complex tools

We have further converging evidence for a special role of right PFt^15,60,61^ in performance of complex and demanding manual tasks. While its partial contribution to haptically guided grasping of tools was revealed in concert with the nearby PF activity^15^, we previously showed that only right PFt, but not PF, is involved more in the control of finger movements during manual exploration of novel complex objects. Critically, right PFt is later *reactivated* during haptically guided grasping of such complex objects.

Macaque and capuchin monkeys are also capable of performing complex digit movements, including difficult precision grips^62,63^, similarly to humans^64^ controlled by their PPC and fine-grained somatosensory representations of individual digits (fingers). Yet, while monkeys can easily use relatively simple tools, they require quite long training to master the use of complex grippers^2^ for grasping. Perhaps their limitations in performing complex motor-to-mechanical transformations (distalization of the end-effector from digit to tool)^7^ can be explained by the lack of a similarly specialized area PFt, with a putative MIP-PFt projection, and weaker lateralization of functions in their brains.

## Conclusions

In summary, human proficiency in performing compound tasks involving complex tools stems from their abilities to comprehend relations between finger movements and movements of handheld implements, and their translation into relevant actions. We found that the associated processing of multifaced motor-to-mechanical transformations engages more the right rostral inferior parietal cortex. This region of the human brain is specialized in sensory-motor spatial processing which enables finger coordination for skillful handling of complex tools and performance of a wider range of complex actions. These findings may shed some light on the evolution of human brain by inspiring future comparative studies of parietal cortex organization and functioning. Moreover, a better understanding of the right parietal involvement in praxis skills may also contribute to developing more effective neuro-rehabilitation techniques and/or neuroprostheses.

## Methods

### FMRI experiments

#### Materials and methods

#### Participants

Twenty-one native Polish speaking individuals (10 females; mean age = 22.5, SD = 2.21) took part in two counterbalanced experiments testing their right and left hands, respectively. One participant (a male) was excluded from further analyses because of errors in data acquisition that occurred in both sessions (experiments). It is of note that based on our previous research^14,15^, we decided to test a fixed sample size of ~ 20 participants. Therefore, instead of calculating the required number of participants needed to get an effect, we computed the required number of trials per person to find statistically significant differences between our main study conditions in a sample of this size. Only individuals with no history of neurological disorders, normal or corrected to normal visual acuity, and who declared themselves as right-handed were qualified to this study. Participants’ potential contraindications were tested by detailed safety questionnaires, and handedness was verified with the revised version of Edinburgh Handedness Inventory (Mean EHI = 83.5; SD = 10.5)^65,66^. Before taking part in the study, participants were informed about all possible inconveniences linked to participation in this study. They were ensured about their anonymity, and all of them signed a written informed consent form. Furthermore, all partakers were debriefed and reimbursed for their time and efforts. All protocols and procedures used in this project were approved by The Bio-Ethics Committee at Poznan University of Medical Sciences (Ethical Approval No. 63/12), and were carried out in accordance with the principles of the Helsinki 1964 Declaration and its subsequent amendments.

##### Stimuli and experimental setup

Twenty-five real objects were used as stimuli in this project. Twenty of them were functional one-handed tools made of nonmagnetic materials (plastic and/or wood), and five were non-tool graspable objects (i.e., wooden sticks and twigs). The tools were designed in such a way that in half, movements of tools’ effectors were the same as movements of hands and fingers, and in the other half the movements of tool parts were typically in the opposite direction than that of the hand or fingers. The only exception was a *Yankee* screwdriver in which pushing movements of the holding hand translated into rotations of the driver. Thus, there were ten tools associated with a low level of motor-to-mechanical transformations (simple tools), and ten with a relatively high level of motor-to-mechanical transformations (complex tools). Notably, the names of tool categories do not refer to subjectively perceived complexity of actions (participants were equally skilled in using tools from both categories; see Procedure section), but only the kind of computations required to correctly realize actions by given tools. The tools can be grouped into pairs, wherein each pair was grasped similarly and performed the same task, with the only difference being the way in which the goal was achieved. For example, participants were using two kinds of scissors, and the first one required simple closing-hand movements in order to cut (i.e., normal scissors), whereas the second required the opposite, i.e., opening-hand movements to perform a cutting action (i.e., reverse or flipped scissors). Moreover, every tool appeared in two sizes, so there were large and small exemplars of each. In addition to scissors, the whole set of tools included screwdrivers, grippers, bottle-openers and tweezers. All tools and non-tools used in both experiments are depicted in Fig. 1a.

In order to invoke target-directed responses, i.e., appropriate preparation and execution of meaningful actions both with tools and non-tools, we designed the so-called *recipient objects* or action targets (see Fig. 1b). There were three recipients embedded in three walls of the apparatus (14 cm high and 17 cm wide, including side extensions, to more efficiently occlude the other targets) used for their presentation. The three target objects allowed for realization of different actions: e.g., a ray-shaped object protruding from one of the walls was used for simulations of cutting and grasping actions. Before each trial, the best-suited recipient was positioned ahead of the to-be-used tools or, in the case of non-tools, it was randomly chosen for the pointing actions performed with sticks.

As Fig. 1c shows (see also Fig. 1d for overhead view), all stimuli were put on the Velcro-covered surface top installed over participants’ legs, by adapting the apparatus used elsewhere^67^. The positioning of the top was regulated by adjusting its height to the size of each participant’s body (e.g., leg circumference) and adapting its distance to the acting hands, so that the presented objects could be efficiently handled. The stimuli were individually placed by an experimenter in the 45°/135° orientation with respect to the front of the surface top, to make the to-be-handled object easy to recognize, grasp and use^68^. Finally, the positioning of action recipients was also adjusted during consecutive trials, so that the targets could be easily reached and manipulated.

Participants could clearly see all the stimuli via the mirror attached to the neuroimaging coil. Importantly, for making target-directed movements as small as possible, and at the same time for substantially reducing the need for shoulder movements, participants’ elbows were supported by extremity-positioning cushions, which were placed beneath the arms. The home location for the acting hands was by the hips, to which the LU400-Pair response pads (http://cedrus.com/lumina/) were attached with a Velcro belt. Participants’ actions were triggered by auditory/verbal commands delivered via headphones. Trial timing was controlled by SuperLab 4.5.4 (http://www.superlab.com) installed on a MacBook Pro 15.4″ computer (with 2 GHz Intel Core i7 processor and 8 GB 1600 MHz DDR3 RAM).

#### Procedure

##### Training session

All study volunteers undertook intensive two-phase training session before the experiment proper. In the first training phase, the stimuli were used freely, so that participants could understand their mechanics. In the second phase, partakers were asked to perform tool use actions. The training session was concluded only when participants were able to correctly use all tools and when they were equally familiarized with all objects^69^, and potential effects that could be directly linked to tools’ novelty or atypicality^38,70,71^ were eliminated. As previous studies suggest, people are relatively fast in obtaining stable neural representations of tools^11,72^, so the undertaken training should minimize the impact of the above-mentioned confounds.

##### The experiment

Two separate experiments with the use of an event-related paradigm were conducted. All participants completed them on different days using their dominant right and the non-dominant left hands in a counterbalanced order. Before the study, we estimated that at least 50 trials per condition were needed to get significant results with the power of 0.8 (80%). To further ensure we had sufficient power, we even increased the number of trials to 60 per condition. It was possible because we always planned to test the two hands in two separate sessions. Specifically, each experiment consisted of six functional runs, each comprising twenty trials: five trials involved simple tools, five complex tools, five non-tools, and finally there were five longer (16-s) rest intervals. Tools from each category were used 30 times across the whole experiment (i.e., a single session). Each run had a different pseudo-random stimulus order and they were assigned to each participant in a random sequence.

At the beginning of each experimental run, participants were asked to push a button on the response pads attached to their hips, and to keep the button pressed when the tested hand was not engaged in grasping or using stimulus objects. Within each trial, participants followed auditory cues presented in the following order. First, a descending sound signaled to the participants that they should close their eyes. Within a subsequent variable inter-stimulus interval (ISI) of 4.5, 5.0 or 5.5 s, the experimenter put either a tool or control object, together with an appropriate action recipient, on the apparatus surface top. Then, an ascending sound—signaling to the participants to open their eyes—was immediately followed by a verbal “Plan” command. In the case of tools, the command indicated planning a functional grasp in the context of the required subsequent action, regardless of whether simple or complex. In the case of non-tools, the *plan* command indicated preparation for grasping of a stick for subsequent pointing movements. There were no further specific instructions on how the planning should be performed. The length of the planning phase was also variable and included delay time intervals of 3.5, 4.5, or 5.5 s. Following a subsequent “Grasp” command and a variable delay interval of 3.0, 3.5, or 4.0 s, participants released a response button, grasped and then held the presented object. No overt action was allowed until the subsequent “Use” command was delivered, although the post-grasp interval unavoidably involved additional preparation for object usage. Following the *use* command, with either a tool or control object, participants performed a suitable manipulation on an action recipient. This phase had a fixed length of 4.5 s, and participants were asked to use the objects until a “Go Back” cue was delivered, which was then followed by a variable 5.25, 5.75 or 6.25 s time interval concluding a given trial. During these inter-trial intervals (ITI), participants put the stimuli on the table, moved their hands back to home position, pushed a button on a pad and waited for the beginning of the next trial. If no object (but a recipient) was put on the table following an “eye-opening” command, participants’ task was to rest, while looking straight ahead, and to wait inactive for the next trial.

Participants were asked to perform actions as precisely and naturally as possible, while avoiding movements of their shoulders and heads which were immobilized with padding. Their performance was always monitored by both experimenters. One of them was standing by the apparatus and could see the acting hands from the side, as their movements were located outside of the scanner bore. The other experimenter monitored participants’ performance from the control room. If any of them noticed any errors, a given trial was marked for the exclusion from data analysis. There were short breaks between runs during which participants could relax and ask any questions. The overall layout of a single trial is depicted in Fig. 1d.

#### Additional localizer scans

Each participant was also tested twice in a *Tool Use Localizer* (TUL), whose goal was to find a measure of brain activity limited primarily to tool use actions themselves, with little contribution from action planning and object grasping. Specifically, an experimenter handed in the objects to the participants’ right (TUL_R_) or left (TUL_L_) hands and the task was to immediately start using these objects. The stimulus set from a main experiment was used, except for all bottle openers, large grippers, and the longest of the sticks. There were four blocks with the use of simple tools, four with complex tools, four blocks of non-tools and, finally, four rest blocks (sixteen in total). Each block lasted 20 s and there were four different orders of objects per block, with each object randomly replaced by the experimenter following a tone presented every 5 s. One of two pseudorandom orders of all blocks was used on given day. During the rest blocks, there were no objects handed in, and as a result they were not even passively held. The outcomes of TUL were later used for conducting Region of Interest (ROI) analysis.

#### Data acquisition

Scanning was performed using a 3 T MR scanner (Siemens MAGNETOM Spectra) in *RehaSport* Clinic, in Poznań, Poland. The MRI scanner was equipped with a 16-channel head coil for radio frequency transmission and signal reception. For each participant, two standard-resolution T1-weighted anatomical images were acquired using three-dimensional magnetization-prepared rapid gradient-echo imaging (3D MP-RAGE) with the following parameters: time of repetition (TR) = 2300 ms, time to echo (TE) = 3.33 ms, inversion time (TI) = 900 ms, flip angle (FA) = 9°, voxel matrix = 240 × 256, field of view (FoV) = 240 × 256 mm, 176 contiguous sagittal slices, 1.0-mm isotropic voxels. Moreover, one fast-spin T2-weighted anatomical scan was acquired: TR = 3200 ms, TE = 417 ms, FA = 120°, voxel matrix = 256 × 256, FoV = 256 × 256 mm, 192 contiguous sagittal slices, 1.0-mm isotropic voxels (to improve image registration). For all functional runs, T2*-weighted gradient echo sequences were used, with the following parameters: TR = 2000 ms, TE = 30 ms, flip angle FA = 90°, voxel matrix = 58 × 64, Field of View FoV = 181.25 × 200 mm, 35 axial slices with an in-plane resolution of 3.125 × 3.125 mm, and slice thickness of 3.1 mm. Data from each run of the main experiment contained 237 volumes, and 185 volumes in the case of the localizer scans. We used MRI-Convert 2.1 software (http://lcni.uoregon.edu/downloads/mriconvert) to convert raw DICOM (Digital Imaging and Communications in Medicine) files to NIfTI-1 format, prior to data analysis with FMRIB’s Software Library (FSL; http://fsl.fmrib.ox.ac.uk/fsl/fslwiki/).

#### Data analyses

##### Behavioral data

For the response time data, movement onsets were obtained from the releases of start buttons after participants completed action planning and initiate a grasping movement. These data were analyzed, using a 2 × 3 repeated-measures analysis of variance (rmANOVA), with hand (right, left) and object category (simple tools, complex tools, and non-tools) as within-subjects factors.

##### FMRI whole brain analyses

For all planning- and grasp-related time intervals, changes in neural signals were modelled for the shortest of their respective variable intervals, i.e., for 3.5 s in the case of action planning, and 3.0 s for grasp execution, starting from a task-related auditory command. The approach used here is analogical to the ones utilized elsewhere^8,14^. The use-related and resting intervals were always modeled / analyzed through their entire durations, i.e., for 4.5 s and 16 s, respectively. As a result, we had ten explanatory variables (predictors), for complex and simple tools, as well as control objects in each of the study phases, and one predictor for rest intervals. The remaining time intervals (including the longer periods for planning and grasping) were not explicitly modeled, and contributed only to the implicit baseline. Importantly, events in which an experimenter reported any performance error (e.g., an inappropriate grasp) or response anticipations (e.g., a button release before a grasp cue) were excluded from the analyses. Only 0.68% of responses were errors.

All neuroimaging signal processing was performed with the use of FSL v5.0.9^73^. Non-brain tissues from T1- (averaged) and T2-weighted anatomical images were removed with the use of BET algorithm^74^. Spatial normalization of all functional images was performed using an interpolation method based on linear transformations (FLIRT) with default cost function for the following series of steps. First, functional data were co-registered to T2-weighted anatomical scans with 6 degrees of freedom (DOF), then T2-weighted images were aligned to T1-weighted images with 7 DOF, and finally T1-weighted images were registered to the standard Montreal Neurological Institute (MNI-152) 2-mm template brain with 12 DOF.

Statistical analyses of functional data were performed with FSL’s FMRI Expert Analysis Tool v6.00^73^. Preprocessing involved motion correction (MCFLIRT), noise estimation, spatial smoothing using a Gaussian kernel of full width at half-maximum (FWHM) = 6.2 mm, and temporal smoothing using high-pass filtering σ = 45 s. Hemodynamic responses were modeled with a double-gamma function. The analyses were performed in three steps: (1) separately for each experimental run, and (2) averaged across all runs at a participant level using a Fixed Effects model, and subsequently (3) at a group level, but now using a Random/Mixed Effect model: Flame 1 (and for the most critical comparisons, also the more detailed Flame 1 + 2 procedure). The resulting Z (Gaussianized t/F) statistic images were thresholded using the FSL’s settings of Z > 3.1 (p = 0.001), with family-wise error rate (FWER) controlled at α = 0.05^20^.

Finally, for each phase of action, two separate repeated-measures ANOVAs were conducted for data from both experiments. The first 2 × 2 rmANOVA had a hand (left, right) and object category (tool, non-tool) as a within-subjects factors and tested for main effects of hand and object, as well as their interaction. The second 2 × 2 rmANOVA had a hand (left, right) and tool category (simple, complex) as within subject-factors and now tested for a main effect of tool complexity and its interaction with a hand factor. Importantly, these contrasts had rest as reference, that is, inputs to these 2 × 2 rmANOVAs involved comparisons of complex tools vs. rest, and simple tools vs. rest from respective study phases.

##### ROI (region of interest) analyses

In addition to the whole-brain approach, separate analyses of changes in neural activity, as compared to rest intervals, in selected brain areas belonging to PRN (the *praxis representation network*)^56^ were performed. The following left-hemisphere regions were chosen as ROIs: cMTG, PMv, PMd, rMFG, cSPL^8^, and additional five areas belonging to IPL: three subdivisions of the intraparietal sulcus IPS1, IPS2, IPS3 (as defined elsewhere)^53,75^, and cytoarchitectonically defined subdivisions of the supramarginal gyrus, namely PF and PFt^76^. The first two ROIs, i.e., cMTG involved in storing action concepts^57^ and PMv involved in programing of movement kinematics^11^ were chosen a priori. The last one, namely PFt, was selected a posteriori, based on our main findings. The remaining ROIs were utilized for consistency with earlier research^8,15^ (and are reported only in Supplementary materials). To avoid the so-called *double dipping*^77^, the analyses were conducted within 5-mm diameter spheres based on coordinates established from signal peaks associated with simple and complex tool use actions in localizer scans, i.e., obtained separately for each hand. Because the outcomes of TUL showed no neural activity within rMFG above the excepted threshold (Z > 3.1), the analysis was based on coordinates reported elsewhere^14^. For each ROI, FSL’s FEATquery tool was used for calculating mean percent signal changes (vs. baseline), separately for voxels active during planning, grasping and using, and disparate kinds of objects. The results were used to conduct 3 (phase: planning, grasping, using) × 3 (object category: complex tools, simple tools, non-tools) rmANOVAs, separately for each experiment (i.e., for actions performed with the right and left hands). Finally, the neuro/anatomical labels utilized in this report are taken from the connectome workbench atlas^23^ used for the visualization of our outcomes.

### Materials and methods of behavioral experiments

#### Participants

Sixteen participants (11 females; mean age = 21.65, SD = 1.75), 14 right-handed and two left-handed (with handedness verified with the revised version of Edinburgh Handedness Inventory; Mean EHI = 63.4; SD = 59.7)^65,66^, took part in an independent, behavioral experiment performed outside of the fMRI scanner. All volunteers were students, had normal or corrected-to-normal vision and none of them had any history of mental illness or neurological disease. They gave written informed consents for participation. All protocols and procedures used in this experiment were consistent with the approval (No. 63/12) obtained from The Bio-Ethics Committee, and the Local Ethics Committee at Adam Mickiewicz University, and were carried out in accordance with the principles of the Helsinki 1964 Declaration and associated amendments.

##### Stimuli and procedures

The same set of 20 objects (10 simple tools, 10 complex tools) from our fMRI experiments were used. Some of them were now also strengthened by metallic elements (not allowed in the scanner), keeping the size of graspable parts of corresponding tools identical (e.g., complex and simple bottle opener). In every experimental trial, tools were presented on a table with a semicircular notch (limiting movements of the body), 10 cm away from the starting point of participants’ dominant hands. Similar to the fMRI experiments, the stimuli were always placed in the 45° orientation with respect to the front of the table to make grasping with the right hand as comfortable as possible (and flipped appropriately for the left hand). There were six infra-red light emitting diodes attached with a medical tape to thumbs, index fingers and wrists to monitor movement kinematics^78,79^. Three-dimensional locations of these diodes were recorded with the use of the Optotrak Certus motion-tracking system, and timing of trials was controlled by SuperLab 4.5.4. Movement onsets were collected by Cedrus Response Pad RB-840. Vision was controlled by Plato Googles (PLATO Translucent Technologies, Toronto, ON, Canada; Milgram, 1987) synchronized with a desktop computer using National Instruments PCI-DIO24 Digital I/O Card. Each experimental session was preceded by extensive training, wherein participants were familiarized with all experimental stimuli, and the training was concluded only when study volunteers were equally familiarized with all the tools, and were able to proficiently use them.

Each trial began when the index finger and thumb was positioned on the start button, with vision initially occluded with the Plato Googles. After the experimenter put a stimulus tool on the table and initiated the trial, the googles became transparent for a 500 ms preview. Then, either the auditory start cue was immediately presented, or vision was blocked for 2000 ms, and the start cue was presented after such a delay interval. Following the start cue, participants’ task was to appropriately grasp the tool with the dominant hand. Vision was then typically blocked either with movement onset, or simultaneously with the start cue. The adopted paradigm^80^ is shown Fig. 4a. Because no visual feedback was available, grasping was followed by simulated tool use, performed without the recipient objects, and such actions continued until a “Go Back” cue delivered 2000 ms after the start cue. Each participant performed 160 trials, comprising 40 grasp-to-use trials with no delay and with vision occluded only at the moment the start button was released (with movement onset), 40 trials with no delay and with vision occluded simultaneously with the start signal, 40 trials performed with a delay after initial preview, with vision restored again and occluded with movement onset, and additional 40 trials with a delay and vision never restored, even simultaneously with the later start signal. In each condition, half of the trials (20) involved simple tools, and the other half (20) involved complex tools. Similar to the fMRI experiments, participants were asked to perform their actions as fast, precisely, and naturally as possible.

#### Data analyses

Grasp movement onsets were analyzed using a 2 × 2 × rmANOVA, with tool category (simple, complex), trial type (no-delay, delay) and preview (non-occluded, occluded), as within-subjects factors. The maximal distance between index finger and thumb (maximum grip aperture, MGA) during grasping a stimulus tool was calculated for each trial using custom in-house software, and analyzed with an rmANOVA of the same structure.

## Supplementary Information

Supplementary Information.
