# Supplementary material for: Complex tools and motor-to-mechanical transformations
Source: Sci Rep. 2022 May 16;12:8041. doi: 10.1038/s41598-022-12142-3 (PMC9110343; doi:10.1038/s41598-022-12142-3)
Supplement: Supplementary file 1 — Supplementary Information. [file 41598_2022_12142_MOESM1_ESM.pdf]

Complex tools and motor-to-mechanical transformations

Ras, M.[1], Wyrwa, M.[2], Stachowiak, J.[2], Buchwald, M.[1], Nowik, A.M.,[1] Kroliczak, G.[1*]

[1]Action & Cognition Laboratory, Faculty of Psychology and Cognitive Science, Adam Mickiewicz University, Poznan, Poland

[2]Faculty of Psychology and Cognitive Science, Adam Mickiewicz University, Poznan, Poland

ORCID

Maciej Ras https://orcid.org/0000-0002-8193-255X

Michał Wyrwa https://orcid.org/0000-0003-4227-1629

Mikołaj Buchwald https://orcid.org/0000-0001-8764-0032

Agnieszka Małgorzata Nowik https://orcid.org/0000-0002-3872-1685

Gregory Kroliczak https://orcid.org/0000-0001-6121-0536

***Correspondence**

Grzegorz Króliczak, Wydział Psychologii i Kognitywistyki UAM, ul. Szamarzewskiego 89, 60-568 Poznan, Poland

E-mail: krolgreg@amu.edu.pl

Supplementary materials

RESULTS

Right IPL is involved in motor-to-mechanical transformations during the transition from grasping to using of complex tools. The outcomes of additional higher-level mixed-effects analyses with the use of FMRIB's Local Analysis of Mixed Effects (FLAME) stage 1 and stage 2[1].

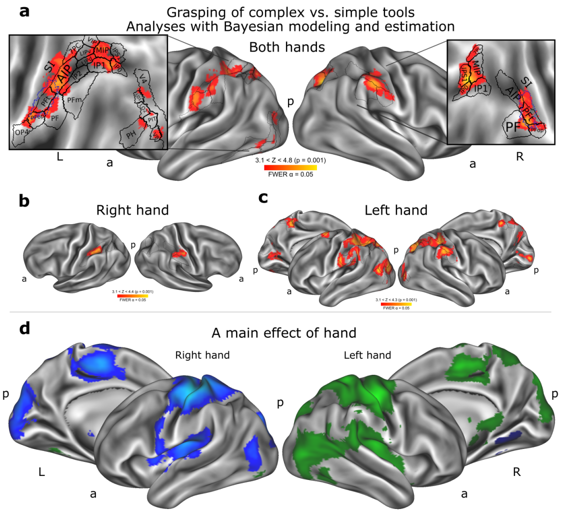

Supplementary Fig. 1 | Neural correlates of motor-to-mechanical transformations revealed by analyses with Bayesian modeling and estimation. a, b, c Task-related contrasts revealing significantly different clusters of voxels, with each voxel thresholded at least at Z > 3.1, and a (corrected) cluster significance threshold of P = 0.05 (controlling for family-wise error rate, FWER), during the transition from grasping to using of complex tools as compared to simple tools (**a**) at hand-independent level, and for dominant right (**b**) and left hand (**c**) separately. **d**, A main effect of hand. The network of areas revealed in this contrast does not overlap with the neural correlates of motor-to-mechanical transformations, and in this way, emphasizes the hand-independence of main results of this study.

Region of Interest (ROI) analyses. The whole brain analysis revealed significant differences between complex and simple tools during grasping/tool use programing, in right PFt and bilaterally superior parietal cortex and Intraparietal Sulcus (IPS). To better understand the modulations of neural activity in the main task, we conducted additional ROI analyses focused exclusively on the activity of left-hemisphere nodes linked to the *Praxis Representation Network*. The coordinates used in these analyses were defined independently based on local peaks of neural signals from our Tool Use Localizer (see Methods). These outcomes are presented in Supplementary Fig. 2.

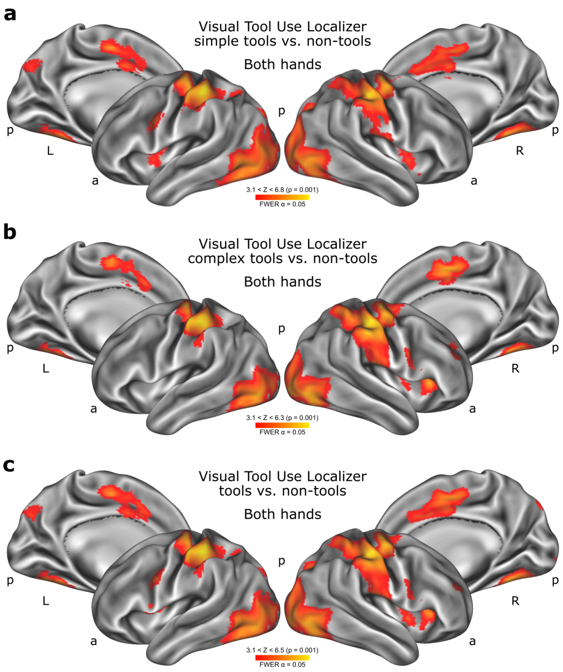

Supplementary Fig. 2 | Brain networks revealed in *Tool Use Localizer,* collapsed across both hands. a, b, c Contrasts revealing significantly different clusters of voxels, with each voxel thresholded at least at Z > 3.1, and a (corrected) cluster significance threshold of P = 0.05 (controlling for family-wise error rate, FWER). Three outcomes are shown: for simple tools **(a)**, complex tools (**b**), and the tool factor collapsed (**c**), in each case as compared with non-tools. To establish coordinates for ROI analyses, more liberal contrasts (i.e., versus rest) were utilized, with the tool factor collapsed (as in [**c**]) but separately for each hand (see Methods).

The less stringent (or more precise) ROI approach revealed, that in fact each area selected for this analysis showed greater responses for complex tools compared with simple tools and non-tools, independently of the studied phase and regardless of the hand (except for cSPL for the left hand). Furthermore, simple tools engaged each ROI more than non-tools, except for IPS1, cSPL and rMFG for the right hand, and PF, IPS2 and PMv for the left hand.

Although most of the brain areas revealed greater responses in the grasping and use phase, independently of the type of object, we also saw significant phase by object interactions. Post hoc analyses for IPS2/PF (right hand), and for cMTG, cSPL, IPS1, and PF (left hand) have shown significant modulations already at the planning phase. Nevertheless, substantially greater increases in activity for complex tools (as compared to both simple tools and non-tools) were observed in the grasping phase in each area, which showed a significant phase by object interaction (cMTG, IPS1, IPS2/PF for a right hand, and cMTG, cSPL, IPS1, IPS3 and PF for left hand). A more detailed summary of the results of the ROI analyses is presented in **Supplementary Table 1**, and bellow.

Supplementary Table 1 | Region of interest analyses. Characteristics of ROIs (MNI coordinates, peak Z values) and the results of statistical analyses (P values for both main effects, and for their interactions).

		MNI coordinates			Peak Z value	Main effect of phase (planning, grasping, using)	Main effect of object (complex tools, simple tools, non-tools)	
Left hemisphere region	Hand	x	y	z				Phase × Object Interaction
Caudal middle temporal gyrus (cMTG)	Right	-46	-64	-2	5.59	0.134	***	*
								planning phase: *** complex tools > non-tools
								*** simple tools > non-tools
								grasping phase: *** complex tools > non-tools
								*** complex tools > simple tools
	Left	-50	-68	-4	4.96	0.255	***	**

								planning phase: *** complex tools > non-tools<br>*complex tools > simple tools<br>**simple tools > non-tools<br>grasping phase: *** complex tools > non-tools<br>*** complex tools > simple tools
Supramarginal gyrus (PF)	Left	-50	-34	50	5.53	***	***	*
								planning phase: *** complex tools > non-tools<br>***complex tools > simple tools<br>***simple tools > non-tools<br>grasping phase: *** complex tools > non-tools<br>*** complex tools > simple tools<br>use phase: *** complex tools > non-tools<br>*** complex tools > non-tools<br>*** simple tools > non-tools
Intraparietal sulcus part I (IPS1)	Right	-30	-48	46	5.14	0.22	***	*
								planning phase: *** complex tools > non-tools<br>grasping phase: * complex tools > non-tools<br>* complex tools > simple tools
	Left	-30	-48	46	3.77	**	***	***
								planning phase: *** complex tools > non-tools<br>***complex tools > simple tools<br>*simple tools > non-tools<br>grasping phase: *** complex tools > non-tools<br>*** complex tools > simple tools
Intraparietal sulcus part II (IPS2) / PF (right hand)	Right	-50	-34	50	5.58	***	***	*
								planning phase: *** complex tools > non-tools<br>* complex tools > simple-tools<br>grasping phase: *** complex tools > non-tools<br>*** complex tools > simple-tools<br>use phase: ** complex tools > non-tools
	Left	-48	-36	52	6.21	***	***	0.068 (trend)
Intraparietal sulcus part III (IPS3)	Right	-36	-46	50	5.36	***	***	0.051 (trend)
	Left	-36	-48	52	4.74	***	***	***
								planning phase: *** complex tools > non-tools<br>** simple tools > non-tools<br>grasping phase: *** complex tools > non-tools<br>***complex tools > simple-tools
Caudal superior parietal lobule (cSPL)	Right	-18	-74	40	5.09	0.066 (trend)	**	0.323
	Left	-22	-74	50	5.38	0.677	*	***
								planning phase: *** complex tools > non-tools<br>**complex tools > simple tools<br>grasping phase: * complex tools > simple-tools<br>use phase: *non-tools > complex tools
Dorsal premotor cortex (PMd)	Right	-30	-12	64	5.17	***	***	0.093 (trend)
	Left	-36	-8	62	5.22	***	***	0.580
Ventral premotor cortex (PMv)	Right	-60	10	24	5.51	***	***	0.067 (trend)
	Left	-52	2	30	4.37	***	***	0.658
Rostral middle frontal gyrus (rMFG)	Right	-36	36	30	-	0.157	***	0.054 (trend)
	Left	-36	36	30	-	*	***	0.775

All analyzed regions (except for cSPL for actions performed with the left hand) were engaged more when actions involved complex tools (as compared to simple tools and non-tools, as well), with activity collapsed across action phases. On the other hand, although the use phase (for IPS2/PF, IPS3, PMd, and PMv for actions performed with the right hand, and for PF, PFt, IPS1, IPS2, IPS3, PMd, PMv for the left hand) and grasping phase (IPS2/PF for the right hand, and PF, PFt, IPS1, IPS2, IPS3, PMd, PMv, rMFG for the left hand) were associated with higher signal responses (as compared to planning), the significant Phase × Object Interaction revealed that it is at the planning and, foremost, grasping phase wherein encoding of motor-to-mechanical transformations takes place. Specifically, for the actions performed with the right hand, activity within cMTG was higher for tools compared with non-tools in the planning phase, and for complex tools – requering motor-to-mechanical transformations – compared with simple tools and non-tools in the grasping phase. For IPS1 the observed significant differences were found for complex tools vs. non-tools during grasp planning, and vs. simple tools and non-tools during grasp execution. Finally, the importance of these transformations for the grasping phase was also revealed for IPS2/PF, where, again, complex tools evoked higher responsens than simple tools and non-tools. Furthermore, during the planning phase, significant differences were observed between all objects categories, and in the case of usage, complex tools engaged IPS2/PF more than non-tools. Similarly to actions performed with the right hand, for the left hand, significant interactions were found again in the case of cMTG, IPS1 and PF. Moreover, for the non-dominant hand, interaction of object and phase was observed for IPS3 and cSPL. For each of these ROIs, tools requiring motor-to-mechanical transformations, i.e., complex tools, were associated with greater activity than simple tools and non-tools in both planning and grasping phases (except for cSPL, where in the grasping phase complex tools engaged this ROI more than simple tools, but not more than non-tools, and IPS3 where in the planning phase complex tools engaged this ROI more than non-tools, but not more than simple tools). Furthermore, simple tools evoked greater signal responses than non-tools in cMTG, IPS1 and PF, when the grasp was planned. Interestingly, for PF and cSPL there were significant differences observed also in the use phase: in SMG complex and simple tools resulted in higher activity than non-tools, whereas in cSPL, conversely, the use of non-tools was associated with greater activity. Asterisks indicate a significant difference with p-value of 0.05 (*), 0.01 (**), or 0.001 (***). Inequality marks in Phase × Object Interaction column indicate the greater neural responses.

Right Hand. Effect of phase. A main effect of phase was significant for: IPS2/PF ($F_{1.538,29.231}$ = 10.892, P = 0.000731, ηp2 = 0.364, 1-β = 0.960), IPS3 ($F_{2,38}$ = 12.727, P = 0.000059, ηp2

= 0.401, 1-β = 0.994), PMd ($F_{2,38}$ = 33.365, P = 4.31×10^{-9}, ηp2 = 0.637, 1-β = 1.0), and PMv ($F_{2,38}$ = 10.309, P = 0.000265, ηp2 = 0.352, 1-β = 0.981). For each of these ROIs, the use phase evoked greater signal changes as compared to planning. In the case of IPS2/PF not only the use phase (BF-P = 0.005226), but also the preceding grasping phase (BF-P = 0.001815) evoked higher responses than planning. Similarly, neural activity in PMd was greater during grasping (BF-P = 0.000021) and using (BF-P = 0.000014) phases, too. Here, the use phase also invoked greater neural responses than grasping (BF-P = 0.020444). Furthermore, the use phase was associated with greater signal changes than both planning and grasping in IPS3 (planning: BF-P = 0.001785; grasping BF-P = 0.014008), and PMv (planning: BF-P = 0.009824; grasping: BF-P = 0.002927).

Right Hand. Effect of object category. In the case of cMTG ($F_{2,38}$ = 72.794, P = 1.0×10^{-13}, ηp^2 = 0.793, 1-β = 1.0), complex tools were associated with higher neural responses than simple tools (BF-P = 0.000195) and non-tools (BF-P = 2.35×10^{-8}), and simple tools engaged this area more than non-tools (BF-P = 3.48×10^{-7}). For IPS1 ($F_{2,38}$ = 21.320, P = 6.19×10^{-7}, ηp^2 = 0.529, 1-β = 1.0) complex tools engaged this area more than simple tools (BF-P = 0.000075) and non-tools (BF-P = 0.000038). For IPS2/PF ($F_{2,38}$ = 114.832, P = 7.79×10^{-17}, ηp2 = 0.858, 1-β = 1.0) complex tool-use actions, again, resulted in higher activity than simple tools (BF-P = 0.000002) and non-tools (BF-P = 3.79×10^{-10}), and simple tools evoked greater responses compared with non-tools (BF-P = 2.90×10^{-8}), as well. In the case of IPS3 ($F_{2,38}$ = 25.155, P = 1.10×10^{-7}, ηp2 = 0.570, 1-β = 1.0) complex tools were associated with higher responses than simple tools (BF-P = 0.000102) and non-tools (BF-P = 0.000034), and moreover simple tools engaged this area more than non-tools (BF-P = 0.021137). For cSPL ($F_{1.449,27.537}$ = 9.153, P = 0.002244, ηp2 = 0.325, 1-β = 0.912), only actions with complex tools evoked higher responses than simple tools (BF-P = 0.000036) and non-tools (BF-P = 0.009189). For PMd ($F_{2,38}$ = 16.649, P = 0.000006, ηp2 = 0.467, 1-β = 0.999) and PMv ($F_{2,38}$ = 19.533, P = 0.000001, ηp2 = 0.507, 1-β = 1.0), complex tools were associated with greater activity than simple tools (BF-P = 0.003908; BF-P = 0.006204, respectively) and non-tools (BF-P = 0.000367; BF-P = 0.000073, respectively), but now simple tools again engaged these two premotor cortices more than non-tools (BF-P = 0.026848; BF-P = 0.013206, respectively). Finally, for rMFG ($F_{1.197,22.742}$ = 15.793, P = 0.000341, ηp2 = 0.454, 1-β = 0.982) complex tool use actions evoked higher responses than actions with simple tools (BF-P = 0.000002) and non-tools (BF-P = 0.000599)

Right Hand. Effect of phase by object category interaction. The significant phase by object category interaction concerned primarily the planning and grasping phases. Specifically, the neural activity in cMTG ROI ($F_{4,76}$ = 3.491, P = 0.011346, ηp^2 = 0.155, 1-β = 0.841) in the planning phase was higher when it was associated with complex tools (BF-P = 0.001112) and simple tools (BF-P = 0.000915) compared with non-tools, whereas in the grasping phase complex tools engaged cMTG more than simple tools (BF-P = 0.014156) and non-tools (BF-P = 0.000285). For IPS1 the significant differences ($F_{4,76}$ = 2.512, P = 0.048560, ηp^2 = 0.117, 1-β = 0.687) observed in the planning phase were only present for complex tools as compared to non-tools (BF-P = 0.000184), and in the grasping phase, again, when compared with simple tools (BF-P = 0.048683) and non-tools (BF-P = 0.021059). Importantly, none of these regions differentiated the object categories during usage. In IPS2/PF ROI, instead, significant differences ($F_{2.270,43.137}$ = 4.269, P = 0.016661, ηp2 = 0.183, 1-β = 0.752) were observed in all three phases. In the planning phase

complex tools engaged this ROI more than simple tools (BF-P = 0.030851) and non-tools (BF-P = 4.06×10^{-7}), and simple tools on top engaged IPS2/PF more than non-tools (BF-P = 0.000152). In the grasping phase, complex tools invoked greater signal changes than simple tools (Bf-P = 0.000006) and non-tools (BF-P = 0.000002). Finally, the activity of IPS2/PF was also higher when complex tools were in use compared to non-tools (BF-P = 0.004266).

Left Hand. Effect of phase. As in Exp. 1, a significant main effect of phase was again observed in PF ($F_{1.482,28.163}$ = 13.453, P = 0.000265, ηp2 = 0.415, 1-β = 0.983), IPS2 ($F_{1.469,27.905}$ = 11.446, P = 0.000694, ηp2 = 0.376, 1-β = 0.962), IPS3 ($F_{1.476,28.045}$ = 16.843, P = 0.000065, ηp2 = 0.470, 1-β = 0.996), PMd ($F_{1.45,27.557}$ = 35.626, P = 2.05×10^{-7}, ηp2 = 0.652, 1-β =1.0), PMv ($F_{1.308,24.859}$ = 18.150, P = 0.000090, ηp2 = 0.489, 1-β = 0.995) and additionally in IPS1 ($F_{2,38}$ = 6.591, P = 0.003488, ηp2 = 0.258, 1-β = 0.888) and rMFG ($F_{1.448,28.276}$ = 4.656 P = 0.026450, ηp2 = 0.197, 1-β = 0.655). All these areas (except for rMFG) evoked higher signal responses during using (PF: BF-P = 0.005611; IPS1: BF-P = 0.016713; IPS2: BF-P = 0.012937, IPS3: BF-P = 0.000561, PMd: BF-P = 0.000010; PMv: BF-P = 0.000285) and grasping (PF: BF-P = 0.000245; IPS1: BF-P = 0.046297; IPS2: BF-P = 0.000516; IPS3: BF-P = 0.000407, PMd: BF-P = 0.000003, PMv: BF-P = 0.000701) as compared to planning. In the case of rMFG, object grasping evoked greater responses than the preceding planning phase (BF-P = 0.039050). Furthermore, significant differences were also observed between neural responses evoked by using and grasping in PMd (BF-P = 0.029091) and PMv (BF-P = 0.013745), where usage of objects was associated with higher signal changes than grasping.

Left Hand. Effect of object category. For cMTG ($F_{2,38}$ = 38.020, P = 8.55×10^{-10}, ηp2 = 0.667, 1-β = 1.0) complex tools evoked greater responses than simple tools (BF-P = 0.003617) and non-tools (BF-P = 3.19×10^{-7}), and also higher activity for simple tools compared with non-tools (BF-P = 0.000145). For PF ($F_{2,38}$ = 62.853, P = 8.89×10^{-13}, ηp2 = 0.768, 1-β = 1.0) complex tools were associated with higher neural responses than simple tools (BF-P = 0.000204) and non-tools (BF-P = 4.70×10^{-8}), and simple tools engaged this area more than non-tools (BF-P = 8.93×10^{-7}). For IPS1 ($F_{2,38}$ = 18.447, P = 0.000003, ηp2 = 0.493, 1-β = 1.0) complex tools engaged this area more than simple tools (BF-P = 0.001174) and non-tools (BF-P = 0.000135). In the case of IPS2 ($F_{2,38}$ = 64.512, P = 6.07×10^{-13}, ηp2 = 0.772, 1-β = 1.0) complex tools were associated with higher neural responses than simple tools (BF-P = 0.000359) and non-tools (BF-P = 1.28×10^{-8}), and simple tools engaged this ROI more than non-tools (BF-P = 0.000001). For IPS3 ($F_{1.425,27.068}$ = 15.148, P = 0.000162, ηp2 = 0.444, 1-β = 0.989) complex tools evoked higher responses than simple tools (BF-P = 0.002728) and non-tools (BF-P = 0.001223). In the case of cSPL ($F_{2,38}$ = 3.645, P = 0.035627, ηp2 = 0.161, 1-β = 0.637) pairwise comparison did not reveal any significant differences. For PMd ($F_{2,38}$ = 10.204, P = 0.000284, ηp2 = 0.349, 1-β = 0.980) complex tool-use actions resulted in higher activity than simple tools (BF-P = 0.021480) and non-tools (BF-P = 0.002333). For PMv ($F_{1.554,29.531}$ = 15.767, P = 0.000072, ηp2 = 0.454, 1-β = 0.995) complex tools were also associated with higher neural responses than simple tools (BF-P = 0.014558) and non-tools (BF-P = 0.000554), and simple tools engaged this area more than non-tools (BF-P = 0.011460). Finally, and consistently with the previous two ROIs, for rMFG ($F_{2,38}$ = 8.378, P = 0.000968, ηp2 = 0.306, 1-β = 0.950) complex tool use actions evoked greater responses than actions with simple tools (BF-P = 0.029310) and non-tools (BF- P = 0.001848).

Left Hand. Effect of phase by object category interaction. A significant Phase by Object interaction was observed not only for cMTG, IPS1, and PF (as for the right hand), but also for IPS3 and cSPL. Likewise, when actions were performed with the left hand, the observed differences concerned mainly planning and grasping phases. For cMTG ROI ($F_{2.757,52.375}$ = 4.412, P = 0.009254, ηp2 = 0.188, 1-β = 0.827) in the planning phase complex tools evoked higher signal responses than simple tools (BF-P = 0.049739) and non-tools (BF-P = 0.000176), and simple tools higher than non-tools (BF-P = 0.031335), whereas in the grasping phase complex tools were associated with higher signal changes as compared to simple tools (BF-P = 0.000109) and non-tools (BF-P = 0.000005). Similarly, for IPS1 ($F_{4,76}$ = 8.718, P = 0.000008, ηp2 = 0.315, 1-β = 0.999) in the planning phase complex tools revealed greater activity than simple tools (BF-P = 0.000356), and non-tools (BF-P = 0.000013), and simple tools greater than non-tools (BF-P = 0.020110). In the subsequent phase, again, higher activity was observed for this ROI during grasping of complex tools as compared to simple tools (BF-P = 0.000246) and non-tools (BF-P = 0.000943) as well. For the PF, as in Exp. 1, significant differences were present in all three phases ($F_{2.715,51.587}$ = 3.236, P = 0.033700, ηp2 = 0.146, 1-β = 0.681). Complex tools in the planning phase evoked higher signal responses than simple tools (BF-P = 0.001287) and non-tools (BF-P = 0.000003), and simple tools evoked greater signals than non-tool objects (BF-P = 0.001394). In the grasping phase complex tools engaged PF more than simple tools (BF-P =.000104) and non-tools (Bf-P = 0.000291). Moreover, when objects were used, complex and simple tools evoked higher signal responses than non-tools (BF-P = 0.000993, BF-P = 0.000226, respectively). For IPS3, the observed differences in neural activity were found in the planning and grasping phases ($F_{4,76}$ = 7.711, P = 0.000029, ηp2 = 0.289, 1-β = 0.996). Specifically, in the planning phase, signal changes for non-tools were lower than for simple tools (BF-P = 0.005246) and complex tools (BF-P = 0.000059), and in the grasping phase complex tools engaged IPS3 more than simple tools (BF-P = 0.000231) and non-tools (BF-P = 0.009650). Surprisingly, a different pattern of responses for this interaction was found in the case of cSPL ($F_{2.586,49.126}$ = 7.667, P = 0.000494, ηp2 = 0.287, 1-β = 0.969). Although in the planning phase complex tools, again, evoked greater neural responses than simple tools (Bf-P = 0.005380) and non-tool objects (BF-P = 0.000168), and in the grasping phase, complex tools resulted in greater responses than simple tools (BF-P = 0.020548), using of non-tools was associated with higher signal changes compared to functional usage of complex tools (BF-P = 0.041062).

Motor-to-mechanical transformations in response time patterns. *Outside of the scanner testing*

A 2 (tool category: complex, simple) × 2 (trial type: no-delay, delay) × 2 (preview: non-occluded, occluded) rmANOVA revealed a significant main effects of tool category ($F_{1,15}$ = 7.420, P = 0.0155, ηp^2 = 0.331, 1-β = 0.723), described in the main text. Furthermore, this same rmANOVA showed a significant main effect of trial type ($F_{1,15}$ = 32.611, P = 0.00004, ηp^2 = 0.685, 1-β = 1.0), wherein somewhat counterintuitively responses in delay trials were consistently faster than in no-delay trials (Mean difference [MD] = 84 ms). A main effect of preview ($F_{1,15}$ = 22.768, P = 0.000245, ηp^2 = 0.603, 1-β = 0.994) was also significant and it was such that trials with vision occluded simultaneously with movement onset cues were again, counterintuitively, faster than trials with vision available through movement onset (MD = 48 ms). We also found a significant trial type × preview interaction ($F_{1,15}$ = 10.837, P = 0.005, ηp^2 = 0.419, 1-β = 0.867) and post hoc testing revealed that the

effect of preview was significant only in trials with no delay, thanks to facilitation of response times with occluded trials (MD = 71 ms).

REFERENCES

1. Woolrich, M. W., Behrens, T. E. J., Beckmann, C. F., Jenkinson, M. & Smith, S. M. Multilevel linear modelling for FMRI group analysis using Bayesian inference. *NeuroImage* **21**, 1732–1747 (2004).
